# Pharmacy academics’ perspectives toward interprofessional Education prior to its implementation in Qatar: a qualitative study

**DOI:** 10.1186/s12909-019-1689-5

**Published:** 2019-07-24

**Authors:** Alla El-Awaisi, Sundari Joseph, Maguy Saffouh El Hajj, Lesley Diack

**Affiliations:** 1 College of Pharmacy, QU Health, Qatar University, Doha, Qatar; 2 Center for the Advancement in Interprofessional Education, London, UK; 3 School of Pharmacy and Life Sciences, Faculty of Health and Social Care, The Robert Gordon University, Aberdeen, Scotland, UK

**Keywords:** Interprofessional education, Pharmacy, Academics, Qualitative, Focus group, Middle East, Qatar, Curriculum

## Abstract

**Background:**

The aim of this study was to explore the perspectives of faculty members and academic administrators, at Qatar University College of Pharmacy, towards interprofessional education (IPE) and collaborative practice by identifying enablers, barriers and resources needed to implement IPE within the pharmacy curriculum.

**Methods:**

A qualitative methodology was employed using focus groups discussions. Two focus groups were conducted, one focus group with faculty members (*n* = 5) and another focus group with academic administrators (n = 5) at Qatar University College of Pharmacy. Focus groups were audio recorded and transcribed verbatim by an independent experienced transcriber and validated by the study principal researcher. Thematic analysis was undertaken to generate key themes and subthemes.

**Results:**

The study participants highlighted a number of enablers and challenges encountered as a result of the initial IPE events, for integrating IPE into the pharmacy curriculum. Many provided recommendations and suggestions for effective implementation of IPE. Analysis of the results focused on three main categories: enablers, barriers and recommendations. Overall, seven major themes were identified: 1) intrinsic enabling factors (initial IPE experiences, cross-appointed faculty, accreditation); 2) extrinsic enabling factors (national policy & legislation and advances in pharmacists’ role); 3) student related benefits (roles & responsibilities and agents for change); 4) student hindering factors (student engagement, perceptions & attitudes and gender issues); 5) partnering academic institutions (logistical issues, familiarity with other curricula and commitment); 6) practice environment (hierarchy, healthcare professionals’ attitude and lack of collaborative practice) and 7) IPE delivery (dedicated structure, IPE curriculum and extrinsic support).

**Conclusion:**

Pharmacy academics had positive perceptions towards IPE suggesting a high level of support and readiness to pursue IPE and an opportunity for pharmacy academics to drive the IPE agenda forward in Qatar. However, a number of challenges were reported. These are important to consider to ensure the development of effective strategies for the integration and enhancement of IPE and collaborative practice.

## Background

In recent years, healthcare systems have become increasingly complex requiring close coordination between members of the healthcare team to enhance collaborative working and promote safe, cost effective and high quality patient care [1–3]. Miscommunication and failure of collaboration can negatively impact the healthcare system and health outcomes, and are primary causes of preventable errors with patients [4, 5]. Recognizing the importance and impact of successful interprofessional collaboration, the World Health Organization (WHO) published a seminal document titled ‘Framework for Action on Interprofessional Education and Collaborative Practice’ in 2010 [6]. In this framework, the WHO strongly advocated the development and integration of Interprofessional Education (IPE) into healthcare curricula. They emphasized the importance of adapting team based collaborative models in different clinical settings to enhance the delivery of healthcare services. One of the key messages echoed in the WHO framework is that health policy-makers should introduce policies and strategies promoting IPE and collaborative practice that are appropriate and applicable for their local challenges and needs. A model that is successfully implemented in one geographical location might not meet the needs of another geographical location with a different cultural context and health system organization. In alignment, with the WHO framework, the International Pharmaceutical Federation (FIP) has published a report entitled: ‘Interprofessional Education in a Pharmacy Context: Global report’ (2015). This report recognizes pharmacy as an essential profession within the interprofessional healthcare team, endorses IPE incorporation into pharmacy education and training, and promotes the importance of collaborative practice [7].

There is no consensus or guidelines of the optimum time to integrate IPE into the curriculum, the amount of content, and the best practices to develop interprofessional faculty [8]. However, there are agreements of shared competencies that students need to acquire before graduation that need to be both achievable and assessable [9]. These competencies are referred to as ‘IPE Shared Core Competencies’. They prepare students to work in healthcare teams and to provide collaborative care upon graduation [8]. One of the early competency frameworks established was the UK Interprofessional Capability Framework (2004) and since then a number of IPE shared competencies and capability frameworks have been developed, including the Canadian interprofessional competency framework, the American core competencies IPE Collaborative, and Curtin University’s Interprofessional Capability Framework in Australia [10–12]. Furthermore, a group of researchers in Qatar have developed IPE core shared competencies that are appropriate for the Qatari context [13].

.In an effort to establish the educational and research infrastructure and build a high-quality health workforce with domestically trained Qatari nationals, Qatar currently accommodates branch campuses of some of the leading universities in North America. These include Weill Cornell Medicine-Qatar (originated in the United States), the University of Calgary School of Nursing (originated in Canada), and the College of the North Atlantic (originated in Canada). In 2007, the College of Pharmacy was established as the only national institution in the country: Qatar University. Qatar University College of Pharmacy is the first and only pharmacy degree college in the State of Qatar and is currently accredited by the Canadian Council for Accreditation of Pharmacy Programs (CCAPP).

A recent systematic review has reported a clear absence of research on faculty perceptions towards IPE [14]. This area is important to explore as both students’ attitudes and faculty attitudes can be perceived as posing barriers towards IPE [15, 16]. Parsell and Bligh argued that although organisational and structural barriers can be very challenging to overcome, it is the attitudinal barrier that might be the most problematic [17]. Faculty members who are uncomfortable with the learning styles of IPE or who may not have enough knowledge or experience about the topic itself could have negative attitudes [15, 16]. This may be the case for pharmacy academics in Qatar who are a heterogeneous group from diverse ethnic and educational backgrounds which may affect their perspectives on IPE and collaborative working.

When IPE initiatives fail, it is usually due to unfamiliarity with roles and responsibilities of other professions, stereotypes, hierarchies, attitudinal biases, and lack of the shared competencies that would be needed for effective collaboration [8, 18]. Other known barriers faced during the implementation and developing stages include timetabling issues/conflicts, time limitations, having unequal numbers of healthcare students, geographical distances, contrasting learning needs, lack of commitment, absence of academic expertise, inequality in assessments, different program lengths, planning and resource difficulties, and lack of institutional support [18–22].

Hence, this research focused on pharmacy academics’ perspectives towards IPE prior to its implementation in Qatar. This research was part of a larger mixed method study investigating pharmacy perspectives of interprofessional education and collaborative practice in Qatar & the Middle East [23, 24]. The first stage of this study was completed through a quantitative survey to explore the attitudes and views towards IPE and collaborative practice of pharmacy academics in the Arabic-speaking Middle Eastern countries. The study findings indicated that pharmacy academics in the Middle East are ready to pursue IPE and willing to integrate it into their curricula [24]. The next stage, which is this research, was an in-depth qualitative exploration of the pharmacy academic perspectives in Qatar.

### Aims

The aims of this study were to:

- Identify enablers and barriers perceived by pharmacy academics in Qatar resulting from integrating IPE into the pharmacy curriculum.
- Identify resources needed to implement IPE within the pharmacy curriculum in Qatar.

## Methods

Study Design.

This was a qualitative study and data were collected through two focus groups. Focus groups were selected as the most appropriate method for the qualitative stage. Focus groups can be very helpful in understanding the perspectives of different groups, assessing their needs and identifying enablers, concerns, challenges, or making recommendations for improvements and future plans [26]. They are considered an opportunity for participants to reflect and listen to other views and experiences and compare them to their own [26]. It is important to ensure that the composition of the group has a dynamic that allows flow of content, stimulates conversation, and increases the speed of information generation. Focus groups encourage participants to address together a topic. The topic could be something that, as individuals, they did not consider before, as in the case of this research [27]. Focus groups allow participants to discuss issues together and probe to further highlight certain perspectives. This can generate useful data that may not have been identified during an interview [28].

### Context

This study was undertaken at the College of Pharmacy in Qatar University. The College of Pharmacy was established in 2007 and has full accreditation by the Canadian Council for Accreditation of Pharmacy Programs (CCAPP). The College of Pharmacy at Qatar University offers a five-year Bachelor of Science in Pharmacy (BSc Pharm) and three postgraduate programs: Doctor of Pharmacy (PharmD), Master of Sciences (MSc Pharm) and PhD in Pharmaceutical Sciences. These programs are delivered in English. The BSc program is currently offered only to female students while the postgraduate programs are offered to both male and female students with plans to offer the BSc program to male students in 2019.

Prior to the data collection of this study, two IPE activities had taken place informally and were based on individual faculty academic’s interests. The first was case based activity on Crohn’s disease between pharmacy and nutrition students [29]; and the second was a case based IPE activity on diabetes and was with nursing and pharmacy students [30]. Additionally, pharmacy students received a didactic lecture on IPE in their first professional year. In the second, third and fourth professional year, interaction with other healthcare professionals was integrated through simulated case scenarios in professional skills’ courses and through structured pharmacy practical experiences.

### Participants

Participants were identified from respondents in Qatar of the first quantitative survey stage of this study who indicated they were willing to participate in the focus group. The principal researcher sent the invitations to participants by email along with an information leaflet. A reminder email was sent to interested participants a week before the focus group’s scheduled date.

Two focus groups were convened in English, to explore in depth the perceptions and experiences of the different participants concerning IPE and collaborative practice. Participants were grouped on the basis of shared attributes, interactions, and experiences to put them at ease when discussing topics. Homogenous groups with similar characteristics tend to exchange their perspectives more freely than heterogeneous groups do and are able to relate to one another [25, 31, 32]. Pharmacy academics were divided into two groups: Pharmacy faculty members: academics in the clinical pharmacy and practice section with some also working in practice settings, and pharmacy academic administrators who are academics with administrative portfolios at the college.

### Format

A topic guide was developed based on the responses from the first stage of this study, a discussion with the research team and a literature review [24] (Table 1). Focus groups were conducted in the same format to allow for potential comparison between groups during the analysis. Prior to the commencement of the focus group, all participants provided written signed consent. The focus groups were moderated by the principal researcher (AE) and ample opportunities were given to explore further points raised by participants. An independent observer (LD) was present during the focus groups and took detailed notes, observing the group dynamics. Participants introduced themselves and explained how long they had been in their current role and at the university. Each focus group lasted two hours. At the end of the focus group, the moderator and the observer conducted a debriefing session.

**Table 1:** Guiding Questions for Focus Groups

Importance	1. IPE is considered important for students as part of their education, as academics how do you feel about this?
Implementation and opportunities	2. Have you had IPE sessions in your courses, how did it go? 3. What would be an ideal IPE program at the College Of Pharmacy? Where do you think IPE should be incorporated in the curriculum?
Implementation and Barriers	4. What do you think you may find challenging if IPE was implemented within the pharmacy program?
Practice	5. Can you give us examples of working with other health care professionals? How do you feel that works for you? For those who don’t work in interprofessional team, what do you think the benefits might there be if you were working in a team environment? 6. Once the pharmacy student graduate, do you think they will find a collaborative practice?

### Data analysis

Focus groups were audio recorded and transcribed verbatim by an independent experienced transcriber. Transcriptions were verified and validated by the principal researcher (AE). Inductive thematic analysis was undertaken on the transcripts based on six steps: becoming familiar with the data; generating initial codes; searching for themes; reviewing themes; defining and naming themes and finally producing the report [33]. The principal researcher reviewed all the transcripts several times and coded the data and extracted the main emerging themes. A second investigator reviewed the transcripts and the key themes thus strengthening the validation of study results (LD/ SJ). All authors met to discuss the coding, similarities and differences until consensus was reached on the key themes and subthemes.

## Results

**Fig. 1:**
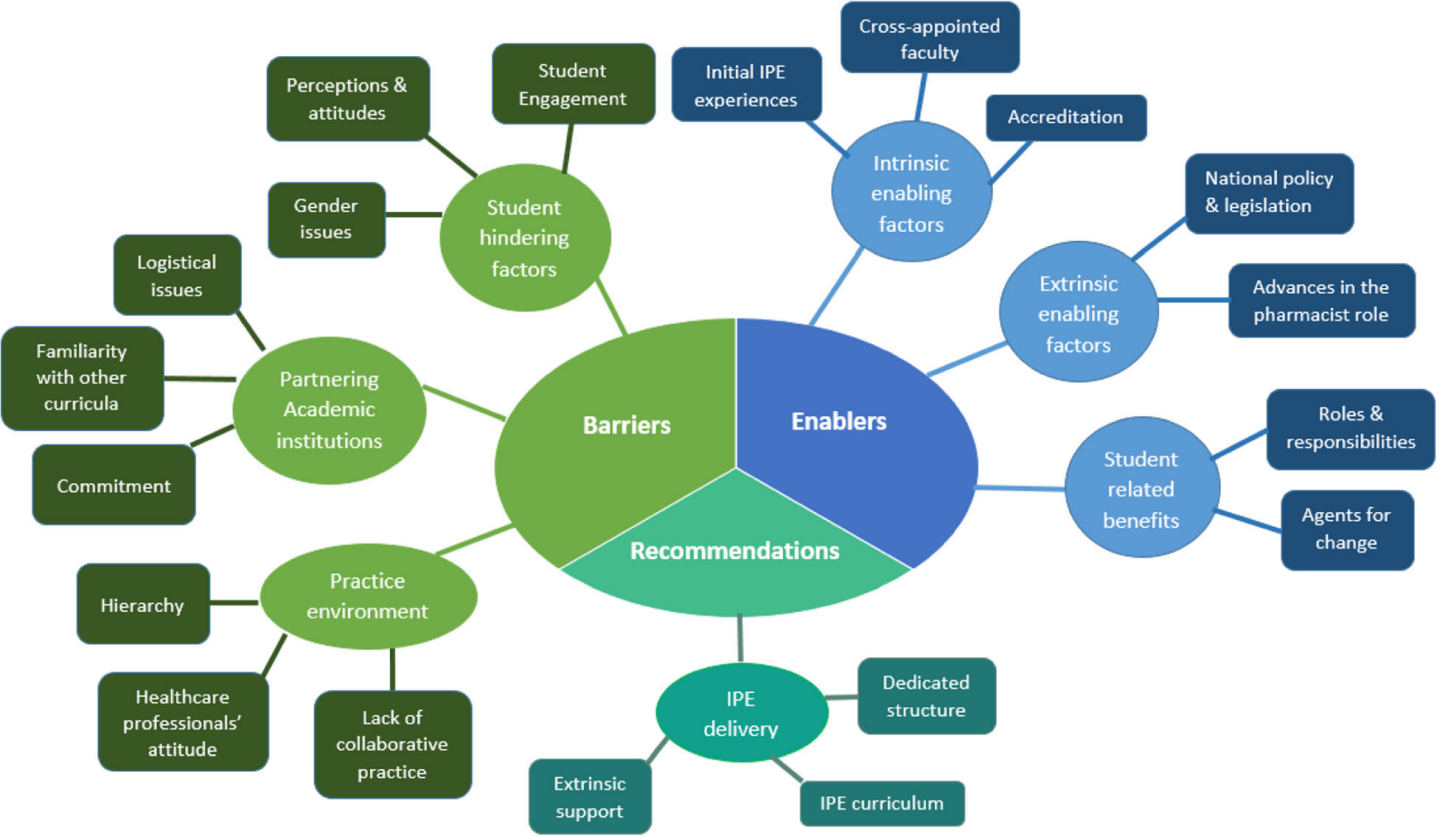
Key Themes and Subthemes for Pharmacy Academics Focus Group

Findings from the analysis are presented under three main categories focusing on enablers, barriers, and recommendations as summarized in Fig. 1. Overall, seven major themes were identified: intrinsic enabling factors, extrinsic enabling factors, student related benefits, student hindering factors, partnering academic institutions, practice environment and IPE delivery.

### Characteristics of participants

The academics who attended the focus groups were: five faculty members from a potential of 10 faculty members and all the academic administrators (*n* = 5). All the faculty member participants (n = 5) were at Assistant Professor level. Their experience working at the College of Pharmacy ranged from 6 months – 5 years. Three of the participating faculty members had joint appointments with a hospital setting. All had a pharmacy background and four were from North America. All had clinical pharmacy experience in a Middle Eastern context. The academic administrators (*n* = 5) who participated in the focus group included the Dean, Associate Dean for Academic Affairs, Associate Dean for Research and Graduate studies, Assistant Dean for Faculty and Student Affairs and the Director of the Doctor of Pharmacy program. Their experience working at the College of Pharmacy ranged from 6 months to 6.5 years.

### Theme 1: intrinsic enabling factors

#### Initial IPE experiences

The evaluation of the activities that had run before this study were well received by academics and students. Faculty members who led these activities recognized the college support that helped to overcome logistic and administrative barriers. Four factors eased the organization of new activities. These were: faculty interest in the topic, prior experience of working with the other faculty members, student enthusiasm, and faculty flexibility to adjust the schedule when needed.

I guess what made it easy was that we had prior relation [with the faculty at University of Calgary], like I knew the person on the other side, before and had worked with them before. That made it easy. I guess the enthusiasm of the students, because it did require modification and movement of their schedule so we had support from other faculty who could switch their lecture time. The students weren’t saying ‘why do we have to go over there? They were open to the experience (Pharmacy administrator participant 5).

The other IPE activity had taken place due to a pharmacy faculty member’s personal interest in the topic from previous experiences:

… I was used to already working with Nutrition. Here in Qatar, they were here on campus, so before we left for the summer, we contacted them and they seemed to be interested (Pharmacy faculty participant 2).

#### Cross-appointed faculty members

Pharmacy academics commented on the College of Pharmacy initiative towards the establishment of cross appointed faculty clinicians where some pharmacy faculty members, in addition to their teaching and academic activities, are assigned clinical duties at Hamad Medical Cooperation to work and precept pharmacy students. The cross-appointed faculty members work closely with other healthcare professionals to provide patient care. Pharmacy academics believed that cross-appointed faculty could play a major role in facilitating IPE especially within the practice settings.

They can use this experience [cross appointment] to like kind of direct how to do this education to fit exactly the real practice. You don’t want like somebody who’s detached from the practice; he doesn’t know exactly the real set up there. So I would think this is a plus initiative we have already the cross appointment (Pharmacy administrator participant 3).

One of the cross-appointed faculty members highlighted the importance of being role models for students:

But we’re hoping that now this year with the PharmD students being precepted by PharmD faculty, they’re actually seeing the collaborative efforts, on our parts, so hopefully they can use that as a model whichever setting that they go to (Pharmacy faculty participant 4).

#### Accreditation

Pharmacy academics felt that having IPE as part of the accreditation standards is a strong driver towards promoting IPE and Interprofessional Collaboration (IPC). They recognized that the college and Qatar University administration have been always supportive of any initiative that is beneficial for students and for accreditation. They also highlighted that IPE is in the college strategic plan and is a priority.

What the university is doing for the programmes so far that they’ve been generous with the resources... especially when it’s anything that’s linked to accreditation, the university is ready to pay money and to make sure that we maintain our accreditation (Pharmacy administrator participant 1).

### Theme two: extrinsic enabling factors

#### National policy & legislation

Participants identified that Qatar is undergoing a slow transition from the traditional physician-centered care to a more team-based care model. They highlighted some of the national initiatives that are ongoing to promote collaboration between the different healthcare professions including developments within the College of Pharmacy. These include the Academic Health System initiative which aims to integrate the health care practices with academia with a focus in its mission and vision on multidisciplinary and collaborative care; the Qatar Simulation Consortium which brings together all the health care professionals and educational institutions in the country with an emphasis on simulation education; the Qatar Interprofessional Healthcare Council which was formed in 2009 with representations from all the healthcare schools in Qatar; and the annual skills competition held by the College of North Atlantic- Qatar. One of the pharmacy faculty members considered these initiatives as a promise leading to a collaborative future:

I am very optimistic to say because most of these initiatives bring together people from all settings including Hamad Medical Corporation, which is the major health care provider in the country. And people from Hamad come and they recognize the value of having pharmacists there, in everything they do and we have been invited in all the initiatives that are happening in the country. So I’m very optimistic about—things will happen. And since there are initiatives in place, I think it will happen soon (Pharmacy faculty participant 5).

Another pharmacy administrator noted that these initiatives are in parallel with other initiatives in the academic settings, which will make transition easier:

so hopefully if these things are happening simultaneously …this will make the change within the hospitals in Qatar easier to happen. So we’re lucky that this is happening here, probably not in other areas in the region (Pharmacy administrator participant 1).

#### Advances in the pharmacist role

One of the participants reflected that the transition of pharmacy practice from the traditional product-centered model to being patient-centered, which makes the development of IPE more important. He highlighted how other healthcare professions have noted the impact of clinical pharmacists on healthcare delivery, leading to more support for teamwork and more interest in collaboration:

… before the concept of clinical pharmacy became clear, we were not really enthusiastic about IPE. Maybe because we did not have much role to play in the wards, in the hospital where the pharmacist were isolated in the basement of the hospital and in some cases there is like a small pharmacy in those like new wards but not working as part of the team, not part of the medical team actually, nor making decision for the patient. This has never been the case. However, things have changed with clinical pharmacists working in the hospital and really more and more doctors are looking are seeking their advice (Pharmacy administrator participant 3).

Similarly, one of the cross-appointed faculty members reflected on her practice experience in a clinic in Qatar where she believed practice is changing slowly:

They’re now beginning, they’ve built a lot of rapport with us, they’re beginning to understand us and now they see what benefit we could give to them. So they are slowly changing their ways, but, it will take time (Pharmacy faculty participant 4).

### Theme three: student related benefits

#### Roles & responsibilities

Pharmacy academics expressed the need for students to learn together as once graduated they will be working together with other healthcare professionals. Therefore, it is essential to gain an understanding about their own contribution to the healthcare team as well as learning about others’ roles and responsibilities, so they can appropriately refer to or interact with other health care providers. This will lead, according to participants, to mutual appreciation and respect.

… as a pharmacist, I do have an important role. I do know things better and there’s an area where I can provide something that the physician cannot. So the physician needs my help in order to better the outcome … And it’s, it’s not the case, so I think we need to build respect and understand, how important for example nursing can be to the health care team, to the patient outcomes and to also understand the limitations of the physician and what role we can provide (Pharmacy administrator participant 1).

Another benefit of IPE for students is that learning in an IPE environment will expand their horizon to allow them to think more collaboratively:

… not get them closed minded, the students. If you’re introducing them to another profession, it kind of expands their mind so it doesn’t just focus solely on what they’ve learned … they would look at the other, the whole picture instead (Pharmacy faculty participant 1).

#### Agents for change

Participants were enthusiastic about what the future holds for their students and foresaw that they will be agents for change:

Since they [pharmacy students] joined this college and we’ve been putting in their mind that ‘you are going to change the practice’ and ‘you are going to change the scope of the pharmacist’ … and this collaboration is going to be part of the change, so I don’t think it’s very far away from the messages that they have been taking and applying over the past years. (Pharmacy administrator participant 4)

### Theme four: student hindering factors

#### Student engagement

One of the highlighted issues is that faculty members indicated that they struggled, at times to engage students from the different professions.

But again you did see a lot of groups where the nutrition and pharmacy were separate, and it was very difficult despite the many facilitators that went to that table to help them, they just, were not mingling very well. Could’ve been a personality issue, or it could’ve been they just probably they did not know how to work with each other in terms of how the other profession would benefit (Pharmacy faculty participant 4).

They acknowledged that some students may have found it easier to focus on the issue from their uniprofessional perspective only.

I think some of the challenges were trying to make that process of facilitating the collaboration between the students and not just having them work in isolation …. In some groups we know it was just easy for them to just work on their problems independently without necessarily coming together (Pharmacy faculty participant 2).

Another faculty member, commenting on a separate activity, felt there was a lack of orientation on how to work together, which led students to cluster in their own profession due to familiarity and comfort, with an element of showcasing their profession as better than the others.

…students weren’t really working together. I felt they were in the same place but they were separated from each other… not talking to each other… … So it was more of being selfish, sorry to say that, more of competition and again I think because from the very beginning it wasn’t structured but because we left it like that, everybody wants to show their strength and be proud of it (Pharmacy administrator participant 1).

#### Perceptions & attitudes

Although students were generally positive about IPE activities, pharmacy academics noted that students may have some perceived negative stereotype that will take time to change.

before they start on and seeing what other professions can do there may be already a hierarchy in their heads… so breaking that down right away and understanding the importance could be something that is a bit difficult right away (Pharmacy faculty participant 1).

Additionally, some of the pharmacy faculty were surprised that some senior students were influenced in the practice setting and were not challenging physicians, although they were capable.

Our PharmD students are very frequently making a recommendation to a patient, and when then we’re like, well why are you recommending that? They say, ‘because the doctor said so- this is what we do’. And they’re not challenging that. They’re not thinking critically themselves (Pharmacy faculty participant 3).

#### Gender issues

Some academics questioned whether the concept of having mixed gender IPE activities is feasible. Some of the pharmacy administrators felt it would not be possible to have mixed gender IPE activities because male undergraduate students are not allowed to enter the female buildings at Qatar University due to Qatari cultural traditions. They perceived that some students find the interactions with male students uncomfortable and some female students may become more passive in certain courses such as physical assessment related courses. However, another academic commented that this is usually student specific. Some are very conservative in their attitudes, but most of the students who go on an internship interview with a male patient and interact with male healthcare professionals have no problems. Academics believed that there should be no segregation in IPE activities as they will be working together when they graduate. The same happens during internship, where they will have to work with all healthcare professionals regardless of gender. Overall, academics believed that this should not be a barrier to integrating IPE but may require more targeted facilitation in the interaction with a focus on both cultural and IPE values.

I think as they go through the years, our students become very confident that I don’t see them having an issue interacting with other male students. I would think maybe in the beginning yes. But towards like their fourth year, especially when they go out into their SPEP (Structured Practical Experiences in Pharmacy) rotations and they’re working with other healthcare providers which the majority of them in Qatar are males, I think they become a little bit more comfortable. (Pharmacy faculty participant 4).

### Theme five: partnering academic institutions

#### Logistical issues

It was apparent from the initial IPE experiences that the diversity of the academic calendars of the different healthcare academic institutions was problematic. For example, Qatar University has two semesters whereas other institutions have three semesters per year. Additionally, participants recognized that IPE activities are more complex and require more time to prepare due to the needs of the different healthcare students. Preparation needed more efforts and coordination, and the collaboration itself took time.

I have a set of learning outcomes for my students that I want to achieve by the end of the two-hour session. Now if I have this mix of students … additional learning outcomes that they want to address so how am I going to manage this so that I don’t have more contact hours with the students. I think this is going to be a critical one, for those who are teaching or coordinating the course I think across all colleges (Pharmacy administrator participant 1).

In addition to attending a number of prior planning meetings between campuses, the geographical distance between the different universities was another reported potential barrier for both the involved students and faculty members as they needed to travel to different locations for the planning and execution of the activity. Although the college arranged shuttle buses for transportation, some academics felt that some students would feel uncomfortable being in an unfamiliar location. Furthermore, scheduling a mutually convenient time in an already heavy and full curriculum was challenging.

#### Familiarity with other curricula

The majority of the participants expressed lack of familiarity with the other healthcare professions curricula in Qatar. Academics who led the initial IPE initiatives noted they learnt about the other healthcare curricula during the process. Another academic administrator was not aware of the healthcare programmes that exist in the country.

we should be exchanging the whole curriculum and exploring where are the areas and which courses do we think we can do things together (Pharmacy administrator participant 1).

Many pharmacy academics in the focus group noted that IPE is a new initiative in the region and hence there is no model in the country or in the region to adopt.

We don’t have a benchmark or a model to follow for example, this means that we need to start by ourselves... I’m sure that we can do it and be the pioneer in it… but this is a challenge of course and we are up to that challenge but it’s not easy (Pharmacy administrator participant 4).

#### Commitment

A significant challenge was the varying levels of interest amongst the different healthcare professions. Although they appeared to be interested, they lacked commitment, as IPE is not a requirement in their curricula. One of the pharmacy faculty members reflected on her experience in the skills competition:

we developed the whole case with very little input from our partnering institution and so the reality is that it’s going to be huge, challenging to do even one-on-one course per year. It’s a huge challenge, so we need to think about all of those issues before going too aggressively and then failing in the process (Pharmacy faculty participant 3).

Another academic administrator highlighted the lack of contribution from the medical school in pursuing IPE opportunities:

if they teach them in a way, that ‘you are the Gods of medicine’ then they will be problematic. But it’s totally in the hands of their mentors and like the administrators of the medical school, how keen they are on IPE. Until now, I don’t see that they want do anything about it (Pharmacy administrator participant 1).

### Theme six: practice environment

#### Hierarchy

Overall, participants felt that the healthcare system in Qatar is still operating on a hierarchical structure. Although IPE was perceived as an important component in overcoming this, it was also felt that these hierarchal differences could impede any initiative, including IPE because of the more traditional attitudes and the current culture. It was also noted that hierarchy not only exists between different professions but also can be within the same profession. This leads to professionals who are perceived to be of a lower status feeling uncomfortable in making recommendations and suggestions:

… there’s a fear of being wrong about something. So I notice like when I’m on rounds at the hospital, they dismiss - if they don’t know the answer to something, they’ll dismiss the concern or the problem as if it’s not an issue. And there’s very little challenge even like for example within physicians. If you’ve had a physician who’s the head- like I’ve seen this happen where if the head of a particular area has showed up on rounds then the physician who’s caring for the patient becomes very passive, and the head of that particular consulting team starts making all the decisions even though they don’t know the patient (Pharmacy faculty participant 3).

A pharmacy administrator reflected on the hierarchical culture in this region, which reinforces the idea that the physician is always at the top of the organizational structure, and this is usually instilled in the mindset of healthcare students. As a result, students, or even healthcare professionals are naturally intimidated by this structure and feel unable to make recommendations or discuss their suggestions.

there are some misconceptions in the society, talking about this part of the world, which I am a part of. And when we look at the, for example the physician, as the doctor, who knows everything, okay, they know everything about drugs. They probably know more than us, I’m just saying what, what a pharmacy student may think, and this will shape their behaviour when they become pharmacists. Being continuously intimidated by the physician if they say something, that they, usually what the physician says is right and is something that cannot be challenged. (Pharmacy administrator participant 1)

There are lots of nurses they’re interacting with [referring to PharmD students], but my impression is… that I don’t perceive that they’re consistently seen as an equal partner in the care provision. …the doctor is at the top of the hierarchy as opposed to the patient being at the top – because we all should be serving the patient (Pharmacy administrator participant 5).

Pharmacy academics, especially those in cross-appointed positions, described situations where nurses are subservient and in many cases, they do not challenge the physician recommendations or requests, and are afraid to speak up because of the negative manner with which they are addressed.

the nurses if they don’t think the patient should get a medication because of something- adverse effect or something -they won’t even tell the doctor, they’ll just say the patient refused it, and just write like ‘refuse’ in the MAR [medication chart] and they won’t approach the physician about it. Because they’re so scared of any repercussions from them--- (Pharmacy faculty participant 2)

#### Healthcare professionals’ attitudes

The healthcare workforce in Qatar is a heterogeneous and international group, from diverse backgrounds and many participants in this focus group have perceived this as a challenge to collaboration, particularly in the physicians’ attitudes towards the advancing role of the pharmacist. Many physicians are accustomed to an environment where they are the sole decision makers and are threatened if another healthcare professional is perceived as challenging their decision.

So imagine as a pharmacist for example coming in and making a recommendation to a medical team, they’re very resistant and very surprised that I would highlight a particular error, or not even error, but something that could be done better. And they feel very threatened by that, so I think that will also come out in IPE sessions as well, because students are being taught by, those health professions (Pharmacy faculty participant 3).

Physicians in particular, still see pharmacists as a threat... They see that maybe pharmacists are embedded and they are encroaching into the areas that are not their areas, so maybe some of the things that need to be done is demystifying this kind of misconception, about some of our role, because sometimes they think when we do these collaborations, it’s trying to encroach into their activities, so there is need to have certain things to demystify this kind of misconception (Pharmacy faculty participant 5).

#### Lack of collaborative practice

Although one of the cross-appointed faculty members commented on his practice as the only model in the country that is ‘very interprofessional and very collaborative’, many noted that in the majority of the hospitals practice is mainly interaction and responding to queries rather than collaboration.

I don’t see a lot of interaction with other healthcare providers. I never see a physiotherapist at the hospital. I never see a dietician at the hospital -I think they exist. I never see a social worker (Pharmacy faculty participant 3).

One administrator reflected on the culture of collaboration:

in this part of the world we tend to be silenced, we don’t tend to work in teams and this is why we try to teach our students to work in teams, although there are negative sides to that but we try to force it (Pharmacy administrator participant 1).

### Theme 7: IPE delivery

#### Dedicated structure

Pharmacy academics, in this focus group, were aware of the complexities of coordinating and planning IPE initiatives. The suggestion of appointing a formal champion to coordinate IPE initiatives was discussed. Others suggested the need for a dedicated structure i.e. establishing an IPE unit or committee with representatives from the different healthcare institutions led by an IPE coordinator and given a budget. This dedicated unit would require administrative support to deal with logistics and organizing the different IPE initiatives. They have noted that although IPE is now an accreditation requirement for many of the healthcare programmes, unfortunately there is a lack of coordinated leadership, which is critically important to develop successful and sustainable IPE initiatives.

I think in terms of coordinating in terms of what will be the systematic delivery of IPE, it needs somebody like the formal champion to coordinate, just know what everybody is doing, to ensure the natural progress of it. So, I think it’s probably, to do it well, it’s insufficient for the course coordinators to work in isolation (Pharmacy administrator participant 5).

Another remit for the dedicated unit would be to organize IPE faculty development workshops to increase awareness about IPE, and the need for it; to learn more about innovative IPE initiatives; to effectively prepare the students for IPE sessions; and to ensure that facilitators are well trained to facilitate IPE activities. Participants felt it is important that faculty members are confident in organizing, leading, and facilitating IPE initiatives across the different healthcare curricula from classroom to practice settings.

We need to train the faculty member to do this, so it’s not only the knowledge that they already have but they need to have skills too, to be able to deliver the right message to students also who are coming from different disciplines (Pharmacy administrator participant 4)

People, I don’t know, maybe they’ll be really excited but don’t know how to implement so things might kind of fall off, or may be resistant to it because they don’t really get it or understand why would it be beneficial for their students. So there would be some education needed with instructors (Pharmacy faculty participant 2).

#### IPE curriculum

Academics agreed that the pharmacy curriculum was already heavily condensed and were not in favor of adding an additional course with more credits specifically focused on IPE. They would prefer to have IPE integrated within assigned courses. Potential courses that were suggested included integrated case-based learning, physical assessment, SPEP, and professional skills. There was even a suggestion of initial shared courses such as pathophysiology, anatomy, physiology, and to some extent pharmacology. The gradual introduction of IPE with vertical integration across the professional years, including graduate programs, was discussed. It was suggested that it could start with theoretical underpinning, then progress to case-based learning, simulation, and finally to integrating the IPE into experiential training, which takes place during the student’s final years.

The question was posed whether introducing IPE too early will ‘dilute the development of their own professional identity’ (Pharmacy administrator participant 5).

Real life cases versus theoretical discussions for IPE experiences were also recommended. A target of one activity per semester per professional year was suggested with one course designated to deliver the IPE activity. Online delivery was not perceived as an option as pharmacy academics felt that face-to-face interaction was an important factor. Additionally, participants hoped that those involved in IPE would be compensated with a reduction in their teaching workload as IPE requires more preparation time.

I think the major concern is just the logistic and the time required, so we did one event in first term and I spent lots and lots of hours just trying to arrange that. And, and then if you incorporate more professions I think that would increase as well (Pharmacy faculty participant 2).

Some participants highlighted the importance of having outreach events and social interaction with other healthcare students to establish relationships that will continue throughout the rest of their career. Others felt that conducting extra-curricular IPE activity would be unrealistic as students already felt overloaded and overwhelmed.

#### Extrinsic enabling support

For IPE initiatives to be successful and sustainable, pharmacy academics felt it is important to align it to the Qatar National Vision and National Health Strategy [35, 36]. They also agreed that support from the university administration and from the Supreme Council of Health [now known as Ministry of Public Health] was deemed necessary for IPE to flourish and advance. Administrators felt that there is a need for sustained and continuous awareness about IPE. For example, one administrator suggested that the Supreme Council of Health through the Qatar Council for Healthcare Practitioners could work on imposing IPC as mandatory for the local accreditation of healthcare practitioners and programs. Another suggested changing the laws that when errors occur, the healthcare team is accountable and liable.

All comes down to buy-in. I think like getting the administration, the faculty and your students on board, plus the other programmes you’re trying to work with and I think all those things will come together. I’ve been involved with other projects now, when you have that buy-in it seems like things do come together but the trick is making sure everyone’s on the same page and realize the benefit (Pharmacy faculty participant 2).

Similarly, participants stressed the need to provide continuous professional development sessions focused on interprofessional practice to practitioners to facilitate and promote sustained collaborative practice.

## Discussion

This study identifies the enablers and barriers for implementing IPE within the pharmacy curriculum in Qatar as perceived by pharmacy academics in Qatar prior to formal integration of IPE into the curriculum. Whilst the pharmacy academics identified challenges, they enthusiastically provided recommendations and suggestions for effective implementation of IPE. Overall, pharmacy academics have recognized the need and importance of IPE inclusion into healthcare curricula which resonates with the positive responses by pharmacy academics in Arabic speaking Middle Eastern countries identified in the quantitative stage, preceding this study. There was a willingness among staff at the different universities to support the integration of IPE into their curricula [24]. This adds to the evidence of positive perceptions to IPE by academics, suggesting a high level of support amongst academics towards IPE [37, 38].

The analysis has also identified similar positive perspectives with faculty members and academic administrators. These results are consistent with those of other studies exploring academic perspectives [38–40]. For example, Lawlis and colleagues conducted a literature review to identify barriers and enablers critical for IPE sustainability and have highlighted five fundamental elements that may inhibit or enhance IPE success and sustainability in healthcare curricula. These include: funding from the government, funding from academic institutions, faculty development programs, support from academic institutions to integrate IPE into healthcare curricula, and commitment by academics from across the healthcare disciplines [40].

Because implementing IPE is an essential component in CCAPP accreditation standards, it has been a key driver and enabler for the incorporation of IPE at the College of Pharmacy. Another important enabler was the opportunity to build on the informal IPE initiatives that had taken place and reflect on the lessons learnt from organizing and implementing these initiatives. These experiences were the foundation for others to collaborate and overcome any potential resistance to change from both academics and the organization [41]. Academics who carried out the initial IPE experiences were motivated and committed to try new initiatives and believed in the value of IPE and collaborative practice. This motivation and commitment leveraged any encountered difficulties. However, sustainability could be threatened if these motivated academics, or even the IPE champion in an institution, were to move or retire, as the IPE momentum may be reduced or lost [41, 42]. Additionally, many may be discouraged if the administration became less supportive of IPE initiatives and are not compensated for their efforts principally by workload reduction or providing other incentives to account for the complexity of designing and delivering IPE initiatives [43, 44].

One of the key challenges highlighted in this study is the lack of collaborative practice in Qatar and the existence of a hierarchical system. This is similar to findings from others studies which focussed on pharmacy students and practising pharmacist perspectives toward IPE and collaborative practice in Qatar [45, 46]. Although IPE was perceived as an important component in overcoming this, it was also felt that these hierarchal differences could impede any initiatives, including IPE because of the more traditional attitudes and culture. The hierarchical structures and stereotyping existing between healthcare professionals can significantly impede IPC leading them to resist the idea of IPE and can have a negative effect on healthcare interaction with pharmacists. Financial differences in salary and salary structure is perceived as a practical barrier to IPE by establishing a class structure detrimental to collaborative practice [50]. These hierarchical issues may result in power struggles between the professions that may be experienced by students undertaking IPE [51]. The powerful global status of the medical profession has been noted as a barrier to IPE success and for these to be overcome, the power differentials between the varied healthcare professions need to be addressed [38, 39].

Academics and health practitioners need to be role models for the students and be able to work with other healthcare faculty members and practitioners to learn with, from, and about each other [47]. However, one study highlighted that academics are fearful that these students will not be able to translate what they have learnt into practice due to the lack of role models in practice to support them in their clinical placement and upon graduation to promote an interprofessional culture [48]. In this study, one of the perceived enablers was the establishment of cross appointed faculty members working between the college and an assigned clinical setting. Although they are intended to support the supervision and evaluation of their own students during their clinical placements and are able to understand and make the connection between education and practice, they can further facilitate the process of translating IPE principles into practice to ensure students have the opportunity to collaborate with other healthcare professionals [49].

An interesting finding in this study relates to the discussion around mixed gender education in IPE activities. Gender segregation in higher education is the norm in most public universities in the Gulf region [45, 52]. At Qatar University, undergraduate education is gender segregated except for the College of Medicine. This is not the case in private universities in Qatar such as Weill Cornel Medicine- Qatar, University of Calgary- Qatar and the College of North Atlantic- Qatar where male and female students are educated together. Qatar University College of Pharmacy currently accepts only female students to its undergraduate programmes, yet students get the opportunity to interact with male healthcare professionals and patients in the clinical training. In this study, academics felt that some students may find interacting with male students to be a challenge but agreed that this is usually student specific and should not be a barrier for integrating IPE in the curriculum. This concurs with pharmacy students’ perspective on the issue where they agreed on the importance of having IPE sessions with male students as they will work in practice with them in the future. They highlighted that it could be perceived as a cultural challenge to some students but with proper orientation and facilitation this could be overcome [45]. Furthermore, academics questioned how an IPE activity could be delivered in a gender segregated building. To overcome this, IPE activities take place in non-segregated campuses such as Qatar University College of Medicine, and private universities such as Weill Cornell Medicine, University of Calgary-Qatar and College of North Atlantic-Qatar.

The study has identified a number of organizational barriers such as the lack of a regional model to adopt, overloaded curricula, logistical barriers, and challenges identified from the initial IPE experiences. Such challenges include the varying level of experiences and knowledge by the students as well as structural differences between the participating institutions such as an incompatible semester timing. One of the key recommendations from this study is the need for a dedicated structure for IPE with a focus on developing an IPE curriculum and faculty professional development workshops. Academic institutions need to facilitate and support the integration of IPE into healthcare and direct resources to IPE for it to thrive. Academics were motivated and enthusiastic during the initial IPE activities, but this may be inhibited subsequently, if they do not feel supported by their management and rewarded for their efforts [44]. In this study, it seems that few healthcare professions remain disengaged or uncommitted to full implementation. Executive leadership and commitment from the different healthcare schools are essential to the development of IPE. If all the schools do not commit equally, academic engagement will vary and resource commitment will be limited [38, 53, 54]. Barriers need to be carefully addressed to develop and sustain an effective and sustainable IPE program and use these as the basis to advance faculty expertise [54]. Moreover it needs to be highlighted that successful integration of IPE requires patience, commitment, long term support, resourcing, provision of incentives and rewards and not over burdening members [42] [21]. Therefore, support in the form of dedicated personnel and allocated finance are critical in ensuring that logistical barriers encountered in implementing IPE and collaborative practice are overcome [39]. This would not thrive without dedicated and committed leadership keen to drive the IPE agenda forward and to declare it as a priority [38, 54].

A positive move is that Qatar University has recently established QU health, a health cluster, bringing the three health related colleges of Qatar University -- Colleges of Health Sciences, College of Medicine, and College of Pharmacy -- under one administrative organizational umbrella to work together and maximize efficiencies. The vision of the health cluster is to: ‘be recognized regionally for excellence in interprofessional health education and interdisciplinary health research; a first choice for students and scholars, and a national catalyst for innovation in the field’ [55]. Therefore, the Health Cluster will serve as a catalyst for IPE, facilitating and strengthening IPE initiatives suited for the Qatari and Middle Eastern context and meeting the highest standard of excellence in the field.

### Strengths of this study

One strength of this study was that it is a part of a larger mixed methods study comprising quantitative and qualitative stages which explored the perspectives of pharmacy academics in the Middle East [24]. It has allowed for further exploration of the pharmacy academics perspective in detail and in a geographical region that has not been investigated before. The findings have already been used as the basis for developing, planning, and leading strategies in the different healthcare institutions to establish, promote, and sustain IPE initiatives and move beyond the traditional healthcare delivery which focuses on achieving profession specific competencies. An Interprofessional Education Committee was formed with several formal IPE initiatives being coordinated through it. IPE is now integrated across the different pharmacy professional years using the University of British Columbia model which is based on three main concepts: exposure, immersion and mastery [56]. As part of faculty development initiative, the committee organized a symposium for academic healthcare faculty in Qatar, in February 2015, to equip them with the knowledge and skills needed to develop and integrate IPE into the different healthcare curricula. This was followed by hosting the First Middle Eastern Conference on Interprofessional Education and collaborative practice under the theme ‘New Frontiers in Healthcare Education’ which attracted more than 300 participants from 13 countries [24].

### Limitations and recommendations

One of the study limitations is that not all pharmacy academics participated in this study as participation was completely voluntary. Those who participated may have been more positive about IPE than those who declined. Another limitation was that only one focus group for every subgroup was conducted and the sample size was small due to the limited number of available participants. Further work is needed to explore the perspectives of other healthcare academics’ attitudes and readiness toward IPE and collaborative practice to ensure a comprehensive understanding of readiness of healthcare faculty to IPE and IPC.

#### Reflexivity

Another important bias to clarify is researcher bias known as reflexivity which demonstrates an awareness on how the researcher’s own bias, belief, value, experience and personal background may have affected data collection, interpretation or even the direction of the research [34]. Although the principal researcher was not involved in the initial IPE experiences discussed in this study, the principal researcher at the time of conducting this study was a clinical lecturer and had worked with the participating academics. This may have affected their responses. However, this may be perceived positively as it may have created a sense of trust. Additionally, it did not deter participants from comprehensively expressing their views at the focus group. The principal researcher was always identified as a PhD student researcher outlining the purpose of the research, using the same standard introduction in the focus group, and assuring participants that no negative consequences would be incurred in the case of non- participation or withdrawal from the study. Furthermore, it is worth noting that prior and during the data collection, neither the principal researcher held an IPE related position nor was she tasked with any IPE related activities.

## Conclusion

In this study, the College of Pharmacy academics’ perspectives towards IPE was investigated qualitatively to determine the strengths and challenges for this group with recommendations on how to overcome the challenges. Pharmacy academics had positive perceptions to IPE suggesting a high level of support and readiness to pursue IPE and an opportunity for pharmacy academics to drive the IPE agenda forward in Qatar. However, a number of challenges were reported and these challenges are important toexplore and overcome so that strategies for implementation can be developed to enhance IPE and IPC. This study enabled further exploration of the pharmacy academic perspectives in a geographical region that has not been investigated before, taking into consideration the Middle Eastern cultural context. The findings in this study have been disseminated and implemented as the basis for developing a successful IPE program in Qatar.

## Data Availability

The dataset used supporting the results of this study are available from the corresponding author on reasonable request.

## Abbreviations

CCAPP: Canadian Council for Accreditation of Pharmacy Programs
FIP: International Pharmaceutical Federation
IPC: Interprofessional Collaboration
IPE: Interprofessional Education
WHO: World Health Organization
